# Chemically defined elicitors activate priming in tomato seedlings

**DOI:** 10.1080/15592324.2022.2095143

**Published:** 2022-06-30

**Authors:** Kiran R. Kharat, Raveendran Pottathil

**Affiliations:** Department of Research and Development, Zero Gravity Solutions, Inc., Boca Raton, FL, USA

**Keywords:** *Solanum lycopersicum* L., BamFX, differential gene expression, stress resistance

## Abstract

Tomato (*Solanum lycopersicum* L.) is an important crop that possesses about 35,000 genes. The treatment of plants with elicitors or pathogen attacks causes a cascade of defense reactions. We investigated tomato responses to the BamFX^TM^ solution containing Zn and Cu elicitors and report the results of comparative transcriptome analysis of tomato seeds treated with Zn and Cu elicitors. The seeds were treated with optimum concentrations of Bam-FX solutions and subjected to cold methanolic extraction methods to obtain the secondary metabolites produced within them at different time intervals post-Bam-FX treatment. The metabolite mixture was analyzed using gas chromatography-mass spectrometry (GCMS). In transcriptome sequencing, GO and KEGG analyses revealed that the majority of the DEGs in BamFx-treated tomato was associated with primary and secondary metabolism, plant hormone signal transduction, TF regulation, transport, and responses to stimuli.The secondary metabolites found in the BamFX treated tomato seedlings – Esters of Fumaric acid, Succinic acid etc. The transcript levels of most auxin transporter-encoding genes changed significantly in the BamFX-treated seedlings (e.g., Solyc01g007010.3, a RING-type E3 ubiquitin transferase). The gene Solyc07g061720.3 for Gibberellin 2-oxidase and the Phorbol-ester/DAG-type domain-containing protein (Solyc02g068680.1) associated with the intracellular signaling genes were found upregulated in the BamFx-treated seeds. The time-dependent effect of the BamFX (1:500 for 60 min) was found to be regulating Abscisic acid signaling pathway genes (Solyc09g015380.1). This study identified many candidate genes for future functional analyses and laid a theoretical foundation for an improved understanding of the molecular mechanisms involved in the BamFx treatment of tomatoes to improve stress resistance.

## Introduction

Tomato (*Solanum lycopersicum* L.) is an important crop model system. The tomato genome possesses about 35,000 genes; a rich resource available to scientists.^1^ The basic chromosome number of tomato is 2 n = 24, and wild forms range from diploids to hexaploids.^2^ An elicitor triggers a hypersensitivity response in plants. Elicitors are very diverse molecules with wide chemical diversity, except that they all trigger the hypersensitivity response.^3–5^ An elicitor’s initial binding to a receptor in or on the surface of the host plant cell triggers the hypersensitivity response by inducing some chemical pathways. The plants’ cell has receptors for elicitors. The specific nature of these receptors is unknown. The mechanism of binding of the elicitor to the receptor that triggers the hypersensitivity response has not been clearly understood. Presumably, a signal transduction mechanism is activated by elicitor-receptor binding. This signal transduction pathway might involve calcium ions, and it is similar to the signal transduction pathways shown to be involved in some hormonal responses.^3,6,7^

During aging and plant degradation, endogenous elicitors can be produced; they include reactive oxygen species (ROS), oligosaccharide, and protein fragments,^8^ also substances generated inside the plant cell, such as hormones (e.g., Jasmonic acid, Salicylic acid), galacturonide, and alginate oligomer.^9^ Another type of elicitor is exogenous substances unrelated to the composition of plants. These are anabolic products of the pathogen that trigger the defense responses of the plant; these may be constituents of the outer membrane, cell wall, or can be excretions.^10^

The treatment of plants with elicitors or pathogen attacks causes a cascade of defense reactions; these reactions include an accumulation of a range of plant-defensive secondary metabolites in intact plants.^10,11^

The regulation of metabolic pathways by multigene families at transcriptional and translational levels leads to activation or inhibition of various signaling pathways. The pathway genes are involved in the production of anti-microbial compounds as well as signaling molecules. The induction of the metabolic pathway has led to the identification of a novel plant defense system for which various mechanisms have been proposed, including salicylic acid and anti-microbial mediated compounds.^1,2,11^

Elicitors induces protein expression of enzymes for the detoxification and phosphate degradation, membrane transports, transcription factors and signal transduction. The proteins from chloroplast, plasma membrane and cell wall are repressed by elicitors.^12–14^

We investigated tomato responses to the BamFX^TM^ solution containing Zn and Cu elicitors and report the results of comparative transcriptome analysis of tomato seeds treated with Zn and Cu elicitors. The goals were to (i) construct a tomato seedling transcriptome; (ii) compare and analyze the transcripts in control and Zn and Cu elicitor-treated plants, and (iii) gain insight into stress tolerance and pathogen-resistance induced by Cu and Zn in tomatoes. This study presents the transcriptome of tomato leaves responding to Zn and Cu elicitors and provides a genetic resource that can be used for crop improvement.

## Results

### Tomato seeds germination in the presence of Bam-FX

Germination of tomato seeds was observed in the presence of BamFX dilutions. Table 1 describes the effect of the BamFX 1:500 dilution (30 min) on the germination of the tomato seeds. The germination rate was 60% in tomato seeds after 48 h and increased to 94% after 72 h. When the seeds soaking time was increased up to 60 min in BamFX 1:500, the germination rate increased up to 68% after 48 h (Table 1).

**Table 1.** Germination percentage of tomato seeds treated with BamFX vs untreated control seeds.

BamFx dilution	Duration of exposure	Germination percentage after 48 h	Germination percentage after 72 h
BamFX 1:500	30 min	60%±2.4%	94%±1.5%
BamFX 1:500	60 min	68%±2.5%	96%±1.5%
BamFX 1:1000	30 min	70%±2.5%	96%±1.5%
BamFX 1:1000	60 min	70%±2.5%	96%±1.5%
Untreated control	-	42%±2.5%	64%±1.5%

When seeds were treated with BamFX1:1000 for 30 min, 70% of the seeds germinated after 48 h, increasing to 96% after 72 h (Table 1).

### Secondary metabolites analysis by using GCMS

We used GCMS/MS for the analysis of the secondary metabolites from the tomato seeds treated with BamFX and untreated control. The secondary metabolites found in the BamFX treated tomato seedlings – Esters of Fumaric acid, Succinic acid, thiocyanic acid, octadecanoic acid, benzoic acid, hexenoic acid, heptanoic acid, Nicotinic acids, carbamic acid and Diethylmalonic acid.

**Figure 1.**
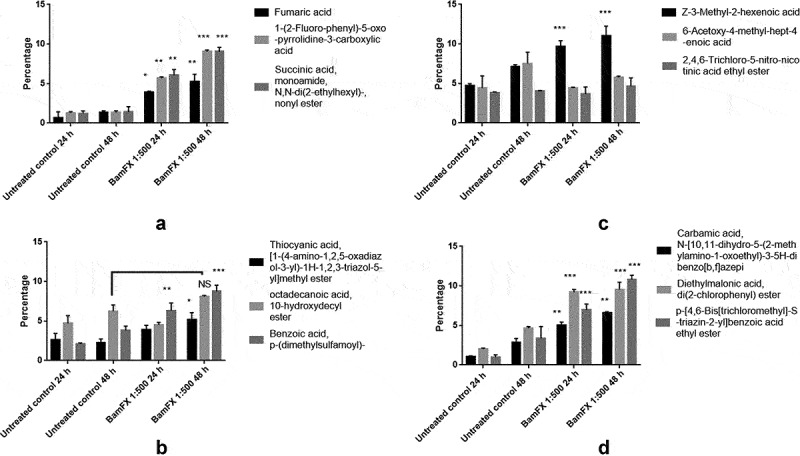
Analysis of secondary metabolites of tomato seedlings by GCMS/MS. Where, Untreated control 24 h – Untreated Tomato seeds incubated in sterile petridish for 24 h,Untreated control 48 h – Untreated Tomato seeds incubated in sterile petridish for 24 h, BamFX 1:500 24 h – Tomato seeds treated with BamFX 1:500 for 30 mins and incubated in sterile petridish for 24 h, BamFX 1:500 48 h – Tomato seeds treated with BamFX 1:500 for 30 mins and incubated in sterile petridish for 48 h. Data are expressed as the secondary metabolites in seeds, where significance refers to the differences between BamFX treated and control untreated seeds (n = 3; ****P < .0001, ***P < .001, **P < .01, *P < 0 .05, ns-not significant). Error bars indicate SD.

**FA, OPCA, SA, TA, ODA, DMSBA, CA, DMA, TCMBA** were found induced in the BamFX 1:500 treated seeds after 24 h of growth (Figure 1). Z-3-Methyl-2-hexenoic acid and 6-Acetoxy-4-methyl-hept-4-enoic acid were found decreased in the BamFX1:500 treated seeds than untreated control seeds.

### RNA-Seq data analysis

To explore differences in the molecular mechanisms of the defense between BamFX (elicitor treated) and untreated control tomato seedlings, we used Illumina sequencing technology to analyze the transcriptome profiles of the seedlings. A total of 2,35,58,528 raw reads were obtained. Approximately 2,29,54,544 clean reads with >95% Q30 bases (those with a base quality greater than 30) were selected as high-quality reads for further analysis (Table 1). The high-quality reads were mapped to the reference tomato transcript sequences, resulting in the mapping of approximately 96% of the nucleotides. Mapping revealed that transcripts of 18395, 18610, and 18229 genes were detected in the BamFX 1:500 and BamFX 1:1000 treated and untreated control seedlings, respectively.

### Functional annotation and classification of DEGs

**Figure 2.**
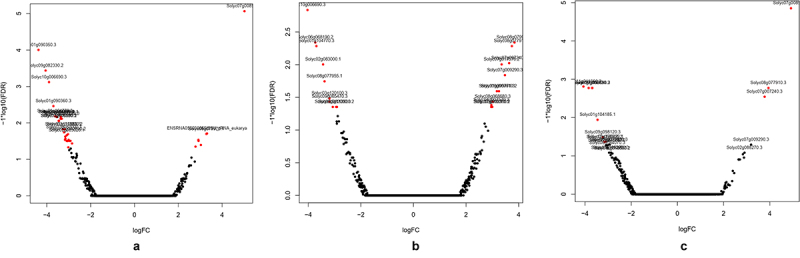
Volcano plot for all expressed genes in BamFX treated seeds-Vs- Untreated seeds. Where- A) Tomato seeds treated with BamFx1:500 for 30 min. B) Tomato seeds treated with BamFX1:500 for 60 min. C) Tomato seeds treated with BamFx 1:1000 for 30 min.

To identify the DEGs between the control (untreated seedlings) and BamFX-treated seedlings, we employed a general chi-squared test with false discovery rate (FDR) correction and a p-value of 0.05 using DEseq6 software to identify two-fold upregulated and two-fold down-regulated genes. In total 2016 genes, significantly DEGs were detected between the control and the treatment samples, with 1142 upregulated genes and 874 downregulated genes being detected in the BamFX samples (Figure 2).

In search of the possible functions of the Differentially expressed genes, local alignment search by BLAST for non-redundant proteins (NR), nucleotide sequences (NT), Clusters of Orthologous Groups (COG), UniProt, gene ontology (GO), and Kyoto Encyclopedia of Genes and Genomes (KEGG) databases were performed.

### GO enrichment analysis of Differentially expressed genes

**Figure 3.**
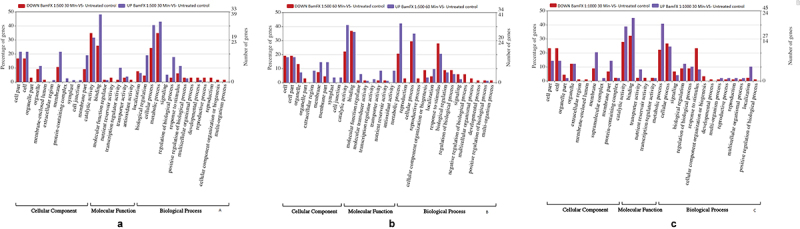
The genes expressed in the tomato seeds treated with BamFX dilutions. A) Tomato seeds treated with BamFx1:500 for 30 min. B) Tomato seeds treated with BamFX1:500 for 60 min. C) Tomato seeds treated with BamFx 1:1000 for 30 min.

Based on the functions of each DEG, a GO enrichment analysis was performed. All the DEGs were grouped into more than 33 functional groups distributed into three main categories: cellular components, molecular functions, and biological processes (Figure 3). The GO functions were significantly enriched in the BamFX-treated seedlings.

The ‘organelle’, ‘cell part’, and ‘membrane terms’ from the cellular components category were significantly enriched. The ‘cellular processes’, ‘response to stimulus’, metabolic processes, and ‘biological regulation’ from the biological processes category were significantly enriched. Whereas from the molecular functions category, ‘catalytic activity’ and ‘protein binding’ were significantly enriched.

Also, several DEGs were classified into two functional subclasses involved with transcription regulator activity and transporter activity. Thus, the majority of the identified DEGs were responsible for fundamental processes associated with biological regulation and metabolism (Figure 3).

### KEGG enrichment analysis of DEGs

To group, the biological functions of the DEGs, a KEGG pathway enrichment analysis was performed. All the DEGs were analyzed by KEGG pathways. Most of the DEGs were protein processing in the endoplasmic reticulum and plant hormone signal transduction along with the photosynthesis proteins, biosynthesis of amino acids, mitogen-activated protein kinase (MAPK) signaling pathway, and carbon metabolism.

### Analysis of DEGs between BamFx-treated and untreated seedlings in the plant hormone signal pathways

In BamFX-treated (1:500 for 30 min) seeds, changes in genes associated with the Jasmonate acid pathway were identified. The significant upregulation of genes in the Jasmonate acid signaling pathways is associated with pathogen infection, plant hormones, and wounding. Solyc12g009220.2 was upregulated in BamFx-treated plantlets.

Most genes associated with the regulation of diverse hormones were differentially expressed between BamFx-treated and untreated seedlings. The transcriptome analysis showed that the expression of genes associated with protein ubiquitination changed significantly. We speculated that these hormone signaling pathways might be involved in differences between BamFx-treated and untreated tomato seedlings.

**Figure 4.**
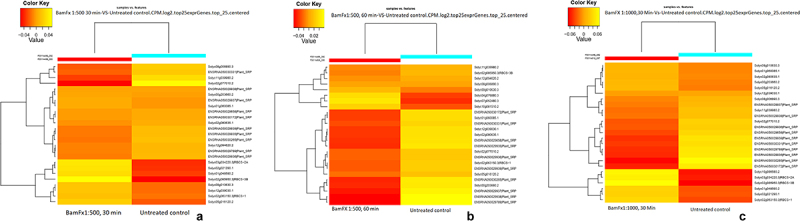
Hierarchical clustering of top 25 expressers. Where A) Tomato seeds treated with BamFx1:500 for 30 min. B) Tomato seeds treated with BamFX1:500 for 60 min. C) Tomato seeds treated with BamFx 1:1000 for 30 min.

The transcript levels of most auxin transporter-encoding genes changed significantly in the BamFX-treated seedlings (e.g., Solyc01g007010.3, a RING-type E3 ubiquitin transferase). The gibberellin is important to enhance cell elongation and induce cell division. The gene Solyc07g061720.3 for Gibberellin 2-oxidase was upregulated in the BamFx-treated seedlings. The Phorbol-ester/DAG-type domain-containing protein (Solyc02g068680.1) associated with the intracellular signaling gene was upregulated in the BamFx-treated seeds. Also, we identified six upregulated genes involved in the protein kinase activity signaling pathways in the BamFX-treated seedlings. Solyc01g095770.3 (involved in ion channel activity) was upregulated (Figure 4).

The time-dependent effect of the BamFX (1:500 for 60 min) was found to be regulating many signal transduction pathways. Abscisic acid signaling pathway genes (Solyc09g015380.1) were upregulated in BamFX-treated (1:500 for 60 min) plants. Many signal peptides (Solyc12g049170.2, Solyc12g049150.1, Solyc12g049070.1, Solyc09g075410.3, Solyc12g100110.1, Solyc12g100110.1 Solyc12g100080.1, Solyc07g017570.2) were upregulated in BamFX-treated (1:500 for 60 min) plants (Figure 4).

Protein kinases expression was elevated in the BamFX-treated (1:500 for 60 min) plants. Solyc12g036325.1, Solyc04g079710.3, and Solyc03g119340.3 were upregulated at the lower concentrations of the BamFX (1:1000 for 30 min). Auxin signaling pathway genes (Solyc08g021820.3, Solyc02g082450.3) and an inorganic phosphate transporter (Solyc03g005530.1) were upregulated (Figure 4).

### Carboxylic acid pathways

Two important genes were found upregulated in the BamFX-treated (1:500 for 60 min) plants. Aromatic amino acid decarboxylase 1A (Solyc08g068680.3) and Fatty acyl-CoA reductase (Solyc06g074390.3) were upregulated in the BamFX-treated (1:500 for 60 min) plants.

Aromatic amino acid decarboxylase 1A (Solyc08g068680.3) and Cytochrome b561 domain-containing protein (Solyc07g048070.3) were upregulated in BamFX (1:1000 for 30 min) (Figure 4).

### Analysis of oxidative stress genes differentially expressed between BamFx-treated and untreated seedlings

The reactive oxygen species are produced during photosynthesis and respiration. The low production of ROS is under strict regulation of the plant cells. Environmental stress can cause an increase in ROS contents. The antioxidant system in plants can remove excess ROS and maintain normal metabolism. In the present study, the significant expression of several candidate genes associated with ROS scavenging, such as Prephenate/arogenate dehydrogenase (Solyc09g011870.2), Fe2OG dioxygenase (Solyc12g006370.2), and L-ascorbate oxidase (Solyc04g054690.3), supported the differential regulation of oxidative stress mechanisms between BamFx-treated and untreated tomato seedlings (Figures 3 and 4).

When tomato seeds were exposed to BamFx 1:500 for 60 min, peroxidase gene expression was elevated (Solyc01g067870.3, Solyc11g007220.2, Solyc02g014300.2, Solyc02g082090.3, Solyc12g017870.2, Solyc01g009400.3, Solyc01g067860.3, Solyc05g055320.3). This expression profile was not found in seedlings exposed to BamFX 1:500 for 30 min. Other enzymes such as Fe2OG dioxygenase Solyc09g089780.3 were also expressed and upregulated in seedlings treated with BamFx 1:500 for 60 min (Figures 3 and 4).

### TFs in the BamFx-treated tomato seedlings

Members of the complex family of WRKY TFs are associated with the transcription regulation associated with the plant immune system. In this study, the expression of many WRKY TFs was upregulated very significantly in BamFx-treated seeds. TFs are involved in gene regulation strictly connected with responses to stress; therefore, the genetic manipulation of TFs is highly desirable. In the present analysis, four TFs were differentially expressed in the BamFX-treated seedlings (Figure 4).

WRKY family members are also directly involved in abiotic stress signaling and tolerance. For example, WRKY23 (Solyc01g079260) responds to auxin regulation, and WRKY70 participates in the defense response to fungus attacks. The present results supported the broad functions of this TF gene family in tomatoes. AP2/ERF (Solyc12g009240.1) and NAC (Solyc07g066330.3) were upregulated in the BamFX-treated (1:500 for 60 min) seedlings (Figure 4).

### Defense proteins in BamFX-treated seedlings

The expression of defense proteins was upregulated in BamFX-treated (1:500 for 30 min) plants. Solyc12g096920.1, Solyc04g007780.3, and Solyc07g009090.3 were up-regulated in the BamFx-treated seeds. In BamFX-treated (1:500 for 60 min) tomato seedlings, the number of genes upregulated was more than with BamFX-treated (1:500 for 30 min) tomato seedlings; and the defense-related gene expression was found upregulated (Solyc07g009040.3, Solyc12g096920.1, Solyc07g009090.3, Solyc07g009030.3, Solyc07g009100.3, Solyc12g009240.1) (Figure 4).

## Discussion

The results analysis of the enriched GO terms revealed that the DEGs were determined to be associated with the enzymatic regulation of metabolism, stress response and signal transduction. BamFX altered the transcription of genes regulating major mechanisms which includes the rearrangement of cell cycle, cell division and regular metabolic pattern. The BamFX affected the antioxidant defense system by upregulating expression of genes with active regulation of the oxidative stress in plants.

The activation of PAMP leading to PTI is the primary level of response that is induced by plant microbial interaction, whereas the secondary level of response is the induction of ETI by recognition of the effectors secreted within the plant cells by intracellular immune receptors.^15–17^

The MAPK pathway is associated with various mechanisms in plant cells such as the biotic and abiotic stresses, regulation of hormones, cell division and differentiation along with the responses to pathogens and abiotic stresses.^13–19^ In the present study, the biological functions of the DEGs were identified by applying KEGG analysis. The majority of the genes were upregulated in the BamFx-treated seedlings in association with the phytohormones signal transduction and MAPK pathway.^16,18,19^ The expression of a RING-type E3 ubiquitin transferase, an auxin transporter-encoding gene was observed as elevated in the BamFX-treated seedlings. The time-dependent effect of the BamFX was recorded to be regulating many signal transduction pathways. The Phorbol-ester/DAG-type domain-containin, which are associated with the intracellular signaling gene was upregulated in the BamFx-treated plants. Abscisic acid signaling pathway genes and Protein kinases expression were found as elevated at the lower concentrations of the BamFX. The Auxin signaling pathway genes and an inorganic phosphate transporter along with Aromatic amino acid decarboxylase 1A and Fatty acyl-CoA reductase were upregulated. The increased transcript level of the important genes associated with ROS scavenging, such as Prephenate/arogenate dehydrogenase, Fe2OG dioxygenase and L-ascorbate oxidase supported the differential regulation of oxidative stress mechanisms.

In conclusion, BamFX induced resistance priming mechanisms in tomato seedlings. The differential gene expression in BamFX treated seedlings revealed the induction of transcription factors and upgraded signal proteins. The oxidative stress genes, defense protein and hormones were found upregulated in the BamFX treated seedlings. The study reports upregulation of the stress related genes leading to development of disease resistance crop varieties in future.

## Methods

### BamFx composition

Copper Sulfate Pentahydrate (CAS-7758-99-8) 2% (w/v) and Zinc Sulfate Monohydrate (CAS-7446-19-7) (7% w/v) mixed in water and pH adjusted to 1.5 with H_2_SO_4_. The BamFx was diluted to 1:500 and 1:1000 in water for further experiments.

### Seeds treatment with BamFX dilutions and the seed germination rate

Hundred Tomato seeds were taken in each Petri dish. The seeds were soaked in BamFX 1:500 and 1:1000 dilutions for 30 min and 60 min.

After 30 min or 60 min soaking in the BamFX dilutions, seeds were removed from the plate and kept in a sterile Petri dish containing wet tissue paper. The growth of the seeds was observed and recorded. The experiment repeated three times. The germination percentage was calculated by using a formula – GP = seeds germinated/total seeds x 100 . Germination rate was determined by calculating the GP at different time intervals after planting and then plotting these data. (Supporting Table 1 and 2) The seeds grown after 48 h sent to the RNA extraction and sequencing.

### Plant material for RNA-Seq

Seeds of tomato were planted and grown in plastic pots and grown at room temperature. Fifty pots (five seedlings per pot) were used in this experiment.

#### RNA extraction, cDNA library construction, and Illumina deep sequencing

Trizol reagent (Thermo, USA) used for the preparation of total RNA from tomato seedlings. The mRNA was purified and used for the library construction with the Truseq™ RNA Sample Prep Kit (Illumina, San Diego, CA, USA) following the manufacturer’s instructions. The six samples were sequenced on an Illumina HiSeq™ 2000 (Illumina). Each sample yielded more than 12 Gb of data. Sequencing was completed by the Neuberg Biology Lab, Ahmedabad, India.

#### Read trimming and optimization

The sequencing adapters were trimmed for each set of sequencing reads, using SeqPrep (https://github.com/jstjohn/SeqPrep), and then low-quality bases (Solexa/Illumina quality score < 25) of the 3′ ends were trimmed using in-house Perl scripts. The quality reads were used for the mapping analysis against the reference genome sequences (ftp://ftp.solgenomics.net/tomato_genome/annotation/ITAG2.3_release/) using Tophat. The Cufflink was used to assemble all mapped reads. The assembled results and original genome annotations were merged and used for further annotation and differential expression analysis.

#### Mapping reads to the reference genome and annotated genes

Open reading frames (ORFs in all transcripts were predicted using Trinity (http://trinityrnaseq.sourceforge.net/analysis/extract_proteins_from_trinity_transcripts.html). Sequence-similarity Blast searches of these transcripts were conducted against the tomato genome reference, the NCBI NR protein database (http://www.ncbi.nlm.nih.gov/), the Gene Ontology (GO) database (http://www.geneontology.org/), the Search Tool for the Retrieval of Interacting Genes (STING) database (http://string-db.org/), and the Kyoto Encyclopedia of Genes and Genomes (KEGG) database (http://www.genome.jp/kegg/). GO terms for tomato transcripts were obtained using Blast2GO (v. 2.3.5) (http://www.blast2go.org/) with default parameters. COG terms were obtained using Blastx 2.2.24+ in STRING 9.0. Metabolic pathways were analyzed by using Blastx/Blastp 2.2.24+ in KEGG (http://www.genome.jp/kegg/genes.html).

#### Differential expression analysis

The Tophat (http://tophat.cbcb.umd.edu/) and Cufflinks (http://cufflinks.cbcb.umd.edu/) programs provide FPKM (Fragments Per Kilobase of exon model per Million mapped fragments) values within a 95% confidence interval. Differential expression was analyzed and calculated according to the count values of each transcript in the two libraries using edgeR (the Empirical Analysis of Digital Gene Expression in R) software. “FDR < 0.05” and “|log2 fold-change (log2FC)| ≥1” were used as the thresholds for judging significant differences in transcript expression. Transcripts with |log2FC| < 0.25 were assumed to have no change in expression levels.

### Statistics

Data are reported as mean ± S.D. All experiments were done at least three times, and three or more independent observations were made on each occasion. Statistically significant values were compared using one-way analysis of variance (ANOVA) and p-values less than 0.05 were considered statistically significant.

## Supplementary Material

Supplemental Material

## Data Availability

The datasets generated and analysed during the current study are available in the SRA repository. The SRA records will be accessible with the following link: https://www.ncbi.nlm.nih.gov/sra/PRJNA798722 Description of Accession: SRX13833453 - BamFX (1:500 diluted in water) treated Tomato seedlings for 30mins. SRX13833454 - BamFX (1:500 diluted in water) treated Tomato seedlings for 60mins. SRX13833455 - BamFX (1:1000 diluted in water) treated Tomato seedlings for 30mins. SRX13833456 - Untreated Tomato seedlings.

## Abbreviations

FA Fumaric acid

OPCA 1-(2-Fluoro-phenyl)-5-oxo-pyrrolidine-3-carboxylic acid (2-chloro-phenyl)-amide,

SA Succinic acid, monoamide, N,N-di(2-ethylhexyl)-, nonyl ester

TA Thiocyanic acid, [1-(4-amino-1,2,5-oxadiazol-3-yl)-1H-1,2,3-triazol-5-yl]methyl ester

ODA Octadecanoic acid, 10-hydroxydecyl ester

DMSBA Benzoic acid, p-(dimethylsulfamoyl)-

CA Carbamic acid, N-[10,11-dihydro-5-(2-methylamino-1-oxoethyl)-3-5H-dibenzo[b,f]azepi

DMA Diethylmalonic acid, di(2-chlorophenyl) ester

TCMBA p-[4,6-Bis[trichloromethyl]-S-triazin-2-yl]benzoic acid ethylester

## Preprint Publication information

Kiran R. Kharat, Raveendran Pottathil. Chemically Defined Elicitors Activate Priming in Tomato Seedlings, 31 January 2022, PREPRINT (Version 1) available at Research Square [https://doi.org/10.21203/rs.3.rs-1235160/v1].
