# Supplementary material for: Chemically defined elicitors activate priming in tomato seedlings
Source: Plant Signal Behav. 2022 Jun 30;17(1):2095143. doi: 10.1080/15592324.2022.2095143 (PMC9746373; doi:10.1080/15592324.2022.2095143)
Supplement: Supplemental Material [file KPSB_A_2095143_SM2797.docx]

Chemically defined elicitors activate priming in tomato seedlings

Kiran R. Kharat^1^, Raveendran Pottathil^1^*

^1^Zero Gravity Solutions, Inc., Boca Raton, FL 33431, USA.

### Corresponding author

Correspondence to: Raveendran Pottathil

Email. accudx@gmail.com

ORCID ID: [0000-0002-5697-3827](https://orcid.org/0000-0002-5697-3827)

### **Supporting Data**

| **n=** | 100 | **Zero hours** | **24 hours** | **48 hours** | **72 hours** |
| --- | --- | --- | --- | --- | --- |
| **Rep** | **Factor A** | 1 | 2 | 3 | 4 |
| 1 | Control | 0 | 3 | 39 | 22 |
| 1 | Expt1 BamFx 1:500 30 min | 0 | 2 | 58 | 34 |
| 1 | Expt2 BamFx 1:500 30 min | 0 | 3 | 54 | 36 |
| 1 | Expt3 BamFx 1:500 30 min | 0 | 0 | 62 | 33 |
| 2 | Control | 0 | 2 | 42 | 21 |
| 2 | Expt1 BamFx 1:500 60 min | 0 | 7 | 59 | 28 |
| 2 | Expt2 BamFx 1:500 60 min | 0 | 3 | 68 | 27 |
| 2 | Expt3 BamFx 1:500 60 min | 0 | 0 | 68 | 28 |
| 3 | Control | 0 | 1 | 39 | 22 |
| 3 | Expt1 BamFx 1:1000 30 min | 0 | 1 | 74 | 20 |
| 3 | Expt2 BamFx 1:1000 30 min | 0 | 4 | 68 | 26 |
| 3 | Expt3 BamFx 1:1000 30 min | 0 | 0 | 63 | 33 |
| 4 | Control | 0 | 1 | 38 | 26 |
| 4 | Expt1 BamFx 1:1000 60 min | 0 | 1 | 65 | 27 |
| 4 | Expt2 BamFx 1:1000 60 min | 0 | 0 | 71 | 25 |
| 4 | Expt3 BamFx 1:1000 60 min | 0 | 0 | 74 | 25 |

Supporting Data

Table 1. Germination of BamFx treated or untreated Tomato seeds in petridishes.

|  |  | **Data Results** | | | | | | | | |
| --- | --- | --- | --- | --- | --- | --- | --- | --- | --- | --- |
| **Rep** | **Factor A** | G %age | MGT | MGR | U | Z | CV_t_ | GI | CVG | T_50_ |
| 1 | Control | 64 | 3.30 | 0.303 | 1.17 | 0.484 | 16.81 | 20.00 | 30.33 | 2.74 |
| 1 | Expt1 BamFx 1:500 30 min | 94 | 3.34 | 0.299 | 1.08 | 0.507 | 15.55 | 28.83 | 29.94 | 2.78 |
| 1 | Expt2 BamFx 1:500 30 min | 93 | 3.35 | 0.298 | 1.15 | 0.482 | 16.23 | 28.50 | 29.81 | 2.81 |
| 1 | Expt3 BamFx 1:500 30 min | 95 | 3.35 | 0.299 | 0.93 | 0.542 | 14.30 | 28.92 | 29.87 | 2.77 |
| 2 | Control | 65 | 3.29 | 0.304 | 1.09 | 0.515 | 15.86 | 20.25 | 30.37 | 2.73 |
| 2 | Expt1 BamFx 1:500 60 min | 94 | 3.22 | 0.310 | 1.22 | 0.483 | 17.71 | 30.17 | 31.02 | 2.68 |
| 2 | Expt2 BamFx 1:500 60 min | 98 | 3.24 | 0.308 | 1.03 | 0.554 | 15.37 | 30.92 | 30.82 | 2.68 |
| 2 | Expt3 BamFx 1:500 60 min | 96 | 3.29 | 0.304 | 0.87 | 0.582 | 13.88 | 29.67 | 30.38 | 2.71 |
| 3 | Control | 62 | 3.34 | 0.300 | 1.05 | 0.514 | 15.29 | 19.00 | 29.95 | 2.77 |
| 3 | Expt1 BamFx 1:1000 30 min | 96 | 3.23 | 0.310 | 0.90 | 0.634 | 15.87 | 30.33 | 30.97 | 2.64 |
| 3 | Expt2 BamFx 1:1000 30 min | 98 | 3.22 | 0.310 | 1.06 | 0.549 | 15.76 | 31.17 | 31.01 | 2.66 |
| 3 | Expt3 BamFx 1:1000 30 min | 97 | 3.36 | 0.298 | 1.00 | 0.533 | 14.99 | 29.45 | 29.75 | 2.77 |
| 4 | Control | 65 | 3.38 | 0.295 | 1.07 | 0.494 | 15.40 | 19.67 | 29.55 | 2.83 |
| 4 | Expt1 BamFx 1:1000 60 min | 93 | 3.28 | 0.305 | 0.95 | 0.568 | 14.47 | 28.92 | 30.49 | 2.70 |
| 4 | Expt2 BamFx 1:1000 60 min | 96 | 3.26 | 0.307 | 0.83 | 0.611 | 13.53 | 29.92 | 30.67 | 2.68 |
| 4 | Expt3 BamFx 1:1000 60 min | 99 | 3.25 | 0.307 | 0.82 | 0.619 | 13.43 | 30.92 | 30.75 | 2.67 |

Table 2. Calculations of the Germination percentage and Germination data of BamFx Treated Tomato seeds .

Where ,the parameter are -

|  | Parameter description |
| --- | --- |
| G %age | Germination percentage |
| MGT | Mean germination time |
| MGR | Mean germination rate |
| U | Uncertainity of germination process |
| Z | Synchronization index |
| CV_t_ | Coefficient of variation of germination time |
| GI | Germination index |
| CVG | Coefficient of velocity of germination |
| T_50_ | Time to 50% germination |
